# miRConnect: Identifying Effector Genes of miRNAs and miRNA Families in Cancer Cells

**DOI:** 10.1371/journal.pone.0026521

**Published:** 2011-10-26

**Authors:** Youjia Hua, Shiwei Duan, Andrea E. Murmann, Niels Larsen, Jørgen Kjems, Anders H. Lund, Marcus E. Peter

**Affiliations:** 1 Feinberg School of Medicine, Division Hematology/Oncology, Northwestern University, Chicago, Illinois, United States of America; 2 Department of Medicine, Ningbo University, Ningbo, Zhejiang, China; 3 Department of Molecular Biology, Aarhus University, Århus, Denmark; 4 Biotech Research and Innovation Centre and Center for Epigenetics, University of Copenhagen, Copenhagen, Denmark; University of Pennsylvania School of Medicine, United States of America

## Abstract

micro(mi)RNAs are small non-coding RNAs that negatively regulate expression of most mRNAs. They are powerful regulators of various differentiation stages, and the expression of genes that either negatively or positively correlate with expressed miRNAs is expected to hold information on the biological state of the cell and, hence, of the function of the expressed miRNAs. We have compared the large amount of available gene array data on the steady state system of the NCI60 cell lines to two different data sets containing information on the expression of 583 individual miRNAs. In addition, we have generated custom data sets containing expression information of 54 miRNA families sharing the same seed match. We have developed a novel strategy for correlating miRNAs with individual genes based on a summed Pearson Correlation Coefficient (sPCC) that mimics an *in silico* titration experiment. By focusing on the genes that correlate with the expression of miRNAs without necessarily being direct targets of miRNAs, we have clustered miRNAs into different functional groups. This has resulted in the identification of three novel miRNAs that are linked to the epithelial-to-mesenchymal transition (EMT) in addition to the known EMT regulators of the *miR-200* miRNA family. In addition, an analysis of gene signatures associated with EMT, c-MYC activity, and ribosomal protein gene expression allowed us to assign different activities to each of the functional clusters of miRNAs. All correlation data are available via a web interface that allows investigators to identify genes whose expression correlates with the expression of single miRNAs or entire miRNA families. miRConnect.org will aid in identifying pathways regulated by miRNAs without requiring specific knowledge of miRNA targets.

## Introduction

Micro(mi)RNAs are small, 19–22 nucleotide long, non-coding RNAs that regulate gene expression mostly by targeting the 3′UTR of mRNAs resulting in reduced translation of proteins or degradation of the mRNAs. miRNAs are fundamental regulators of cell differentiation and developmental processes. They have also been recognized to be highly relevant in cancer formation and progression [1]. Recently it was demonstrated that almost all human genes are under control of miRNAs [2]. However, because miRNAs regulate the expression of hundreds of target genes [3], and many genes are targeted by multiple miRNAs [4], assigning biological functions to miRNAs or miRNA families has been a difficult task.

miRNAs contain at their 5′ end a short stretch of 6–8 nucleotides complementary to the seed match in the target mRNA. This complementarity is accessible to computational analysis and multiple algorithms have been developed to predict miRNA targets [5]. However, target predictions made with these algorithms are not accurate enough to deduce biological function of miRNAs solely based on the lists of predicted targets. Target validation is usually done by either overexpressing miRNAs or by inhibiting their function followed by measuring the changes in mRNA or protein levels in transfected cells [2], [6]. However, both overexpression and inhibition of miRNAs have caveats [7] and it is not clear whether the observed changes at the mRNA and protein level are the result of direct regulation by miRNAs or are the result of changes downstream of the miRNA-targeted genes.

We have recently used the NCI60 cells [8], a panel of 60 cancer cell lines maintained at the NCI, to identify and validate connections between miRNAs and their targets. Unperturbed array data points on expression levels of hundreds of miRNAs and more than one hundred thousand gene probes on multiple array platforms make the NCI60 cells a unique system to identify cancer relevant connections between miRNAs and genes regulated by miRNAs. Using the NCI60 system we previously validated *HMGA2* and *IMP1* as targets of the *let-7* family of miRNAs [9], [10]. In addition, we identified the members of the *miR-200* family and validated two E-box binding transcription factors, *ZEB1* and *ZEB2* as targets [11]. Most recently we validated the tyrosine phosphatase *FAP1* as a *miR-200* target [12]. These examples demonstrate the power of the NCI60 data sets to connect miRNAs with their targets. However, identification of single miRNA targets without a cellular context or knowledge of all the targets for a miRNA makes it difficult to connect miRNAs with biology or pathology. Our studies of *let-7* and *miR-200* in the NCI60 cells also allowed prediction and confirmation of the biological function of these miRNAs. *Let-7* was found to be a marker for differentiated cancer cells [9], [13], and *miR-200* was identified as a powerful marker and regulator of the epithelial-to-mesenchymal transition (EMT) [11], [14].

Most of our findings were made by comparing miRNA and mRNA expression levels, which at the time was surprising, considering that miRNAs in mammalian cells were believed to mostly act by translational silencing without affecting mRNA expression levels. However, it had also been demonstrated that mRNA abundance of the majority of the targeted genes was somewhat affected by miRNAs [15], [16]. While there is still controversy on the predominant way by which miRNAs regulate gene expression [17], a recent study suggested that, for a substantial number of genes targeted by miRNAs, destabilization of mRNA is the main mechanism of protein repression by miRNAs [18], an observation that makes the NCI60 mRNA/miRNA data sets a valuable tool for studying miRNA function in cancer cells.

Transfecting cells with either miRNAs or miRNA inhibitors usually results in changes in abundance of a large number of mRNAs. Interestingly, mRNAs that are negatively regulated by miRNAs are found, as well as a large number of mRNAs that positively correlate with miRNA expression. These changes in mRNA levels have generally been considered to be caused by genes coregulated with miRNAs or to be secondary events. Indeed, it is likely that the majority of genes whose expression responds to changes in the expression of miRNAs are not direct targets of the miRNA *per se*, but are biological effectors relevant to the function of the miRNA. Especially when detected with the steady state system of the NCI60 cells, these biological effector genes may hold important information regarding the endogenous function of miRNAs. This was most obvious for *miR-200*. A small change in *miR-200* expression (about 2 fold) resulted in a moderate change in mRNA levels of its targets *ZEB1* and *ZEB2* (about 5–7 fold), which resulted in a massive change in the *E-cadherin*/*Vimentin* ratio (over 8 orders of magnitude) [11]. The enormous positive correlation between the expression of *miR-200* and the *E-cadherin*/*Vimentin* ratio allowed us to assign the function of “epithelial regulator” to the miR-200 family even before we had identified *ZEB1* and *ZEB2* as targets. Encouraged by this analysis, we have now used the NCI60 system to identify secondary correlators of miRNAs (which can be in the thousands as in the case of EMT [19]) in a genome-wide analysis as they can hold important information on the biological state of a cell.

We have developed a novel method to cluster miRNAs or miRNA families according to their biological correlators rather than their predicted targets or their chromosomal colocalization or their tissue specific expression. miRNAs were clustered into distinct functional groups according to the expression of their biological effector genes. We have validated the activity of one of these clusters, which contains all members of the *miR-200* family, in regulating the epithelial nature of cells. This resulted in the identification of three novel miRNAs, *miR-7*, *miR-203* and *miR-375*, to function in epithelial maintenance. In addition, we have identified clusters of cancer relevant miRNAs that are either growth promoting or growth suppressing in nature based on the correlation of their expression with the expression of either ribosomal proteins or *c-MYC* regulated genes. We have created a web-based interface, miRConnect.org, which provides a robust and easy-to-use tool for investigators to identify novel connections between miRNAs or miRNA families and groups of genes that are markers for various biological states.

## Results

### Generation of correlations between miRNA and gene expression

In order to explore biological activities of miRNA we first established the correlations between expression of miRNAs and genes. We made use of several data sets available for the NCI60 cells (59 cell lines): 1) the expression profile of 208 human miRNAs quantified by real-time PCR (the “Q” data set) [20]; 2) four data sets of human gene expression profiles (STANFORD, GENELOGIC_U95, GENELOGIC_U133 and NOVARTIS) available on the NCI Developmental Therapeutics Program (DTP) server. In the Q data set, 136 miRNAs were defined as being expressed at detectable levels (as assessed by real-time PCR) in at least 30 of 59 cell lines (Table S1). The NCI60 cells represent 9 different cancers. The cutoff of 30 cell lines was chosen to include at least half of cell lines, and to ensure that at least four different tissue origins were represented. An advantage of the NCI60 system is the ability of combining individual endogenous miRNA expression in a way represented by correlating gene expression with the expression of an entire miRNA family (i.e., all 9 let-7 activities represented in the Q data set). The 136 miRNAs contained members of 24 seed families (miRNAs that share the same seed sequence with more than one family member, Table S2). In addition, due to their predicted overlapping function, we generated an additional custom family of all miR-200 family members; miR-200 falls into two different seed families, miR-141/200a and miR-200bc/429, distinguished by only one nucleotide difference in the center of the seed sequence [11], [14].

The most common and well-defined strategy to explore miRNA-gene associations is the Pearson's Correlation Coefficient (PCC) [21], [22]. While the PCC is a powerful tool to detect correlations, it has limitations. For example, PCC gives equal weight to every sample to be measured (e.g. a cell line, a specific tissue or a patient sample). It does not differentiate between samples with high expression and ones with low expression. This may lead to distortion of the correlation analysis, because: 1) the expression level of miRNAs holds important regulatory information; and 2) noise is more likely to exist in samples containing genes of low expression. We therefore thought of a way to overcome some of these limitations. One solution would be to assign different weights to high and low expression levels. However, because the PCC calculation is not a linear process, it is not practical to add weights directly to each sample. Instead, weighting at the level of the sample selection was found to be more practical since PCCs of different arrays of cell line data could be added up and the corresponding modeling would again be linear. Based on this consideration, we developed a novel method, the “summed PCC” (sPCC). A standard (direct) PCC (dPCC), the sPCC and a randomized sPCC (rsPCC) were applied to generate correlations between expression of miRNAs and genes, to test the reproducibility of the correlations and explore the particular biology of how miRNAs work.

### Direct (d)PCC

In this method we performed a standard COMPARE analysis [23], which produces PCCs, to identify mRNAs that correlated with the expression of each of the 136 miRNAs. In this and all subsequent COMPARE analyses we set 30 as the minimal number of detectable cell lines. To normalize the detecting variation among probes respectively included in the four gene array platforms, the PCCs were averaged for each gene. This method gave a PCC value for each mRNA that significantly correlated with the expression of a miRNA.

### Summed (s)PCC

In this method (illustrated in Figure S1) we modified the calculation process of miRNA-mRNA correlation by adding up a series of PCC values mimicking a “titration” of miRNA by ranking cell lines according to their expression of miRNAs. For each miRNA-mRNA pair, we sorted miRNA expression in 59 cell lines from highest to lowest, and selected the top 30 cell lines as the initial condition, because these 30 cell lines represented the top half of all the cell lines and included cells from at least 4 different tissue origins. We performed a COMPARE analysis for these 30 cell lines (pattern 30). Next, the cell line with rank No. 31 was included and a COMPARE analysis was repeated for these 31 cell lines (pattern 31). Repeated COMPARE analyses were performed until all the 59 cell lines were included in an incremental way, and a total of 30 PCC values were generated (pattern 30 to pattern 59). We did not use a sliding window of a fixed size (1 to 30, 2 to 31, …, 30 to 59) because we always wanted to include the cell lines with the highest expression of a miRNA expecting to have the greatest effect on target/effector genes in these cell lines. This additive method assigned weights in a gradient (or titration) way based on miRNA expression levels. The 30 cell lines with the highest expression were always included in each COMPARE calculation and assigned highest weights, since we expected the greatest effects on target/effector genes from these cell lines. The PCC sums were averaged for each gene among the four gene array platforms.

### Randomized summed (rs)PCC

In order to test the stability and reproducibility of the sPCC method, we designed a randomized from of sPCC as an internal control. The only difference between this method and the sPCC method was that cell lines were sorted in a randomized way. For each miRNA-mRNA pair, the randomized sorting was repeated 10 times and the 10 rsPCCs were averaged.

### The sPCC method accurately detects both downstream effector genes and predicted targets correlating with miRNAs

**Figure 1:**
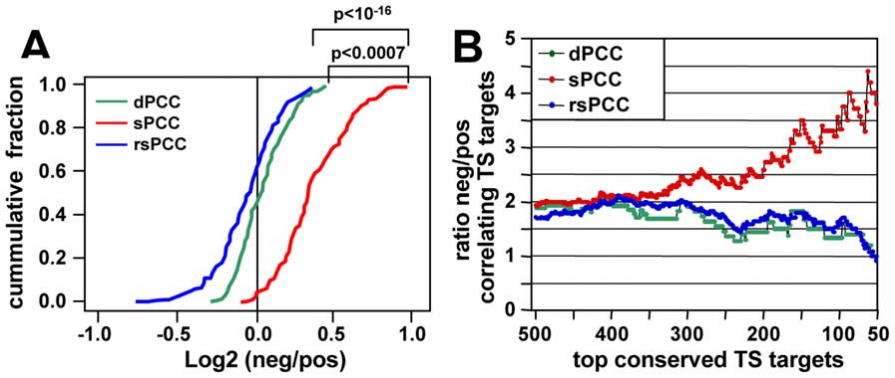
Performance of the NCI60 data sets to detect mRNA/miRNA correlations and to predict miRNA targets. (A) Cumulative plot of log2 ratio of negatively vs. positively correlating gene numbers for 136 miRNAs calculated with the dPCC, sPCC and rsPCC methods, respectively. The X-axis denotes the log2 ratio values of 136 miRNAs according to their ranking from lowest to highest, and the Y-axis denotes the cumulative fraction of 136 miRNAs. The differences in the cumulative curves were measured by one-sided Kolmogorov-Smirnov Test. (B) Comparison of the three methods to identify the most likely conserved TargetScan predicted targets in the human genome (a total of 33,535 predicted targeting events). Target predictions were ranked by total context score from highest to lowest. Incrementally from top 50 to top 500 miRNA-gene pairs with the highest total context scores, the ratio values of negative vs. positive correlation numbers were calculated and plotted for the three methods.

It is known from multiple studies that although altering miRNA expression levels in cancer cells causes both up and down regulation of genes, miRNAs predominantly work through negative regulation of downstream effector genes [15], [16]. In order to test this observation with our methods, the log2 ratio values of all negative vs. all positive correlations were calculated for 136 miRNAs respectively with the three methods (dPCC, sPCC and rsPCC). A comparison of distribution of 136 ratio values demonstrated that negative correlations significantly outnumbered positive ones in the sPCC analysis but not in the two other analyses (Figure 1A). The log2 ratio with the sPCC method significantly shifted to the right compared to the dPCC method. A higher number of miRNAs in the sPCC method had negative correlating genes, which was canceled out by random noise in the dPCC method (the median value was roughly zero). The cumulative curve of rsPCC was similar to that of dPCC, but was significantly different from that of sPCC. This demonstrates that the sPCC method was more effective in detecting negative correlations than either the dPCC or the rsPCC method.

Next we sought to compare the efficiency of the three methods in detecting predicted targets. We chose TargetScan, a widely used target prediction algorithm, to assemble a list of all human miRNA-gene pairs involving the 136 miRNAs. The list was sorted by total context score (defined by TargetScan for the conserved targets) from highest to lowest (Table S3). Then incrementally from top 50 to top 500 miRNA-gene pairs, the ratios of negative vs. positive correlation numbers were calculated and plotted. Only with the sPCC method, this ratio increased along with the increase of total context score (Figure 1B). Therefore, results for all correlations and the correlations with regard to predicted targets both suggested that the sPCC method performed significantly better at the theoretical level than either the dPCC or the rsPCC method.

### The sPCC method accurately detects expression of miRNAs that are linked to single host genes as well as to clusters of *HOX* genes

In addition to the theoretical level, it was necessary to test the ability of the sPCC method to identify miRNA/gene connections that have been established in known biological systems. We therefore made use of both host genes and homeobox (*HOX*) genes that have well characterized links to the expression of certain miRNAs.

**Figure 2:**
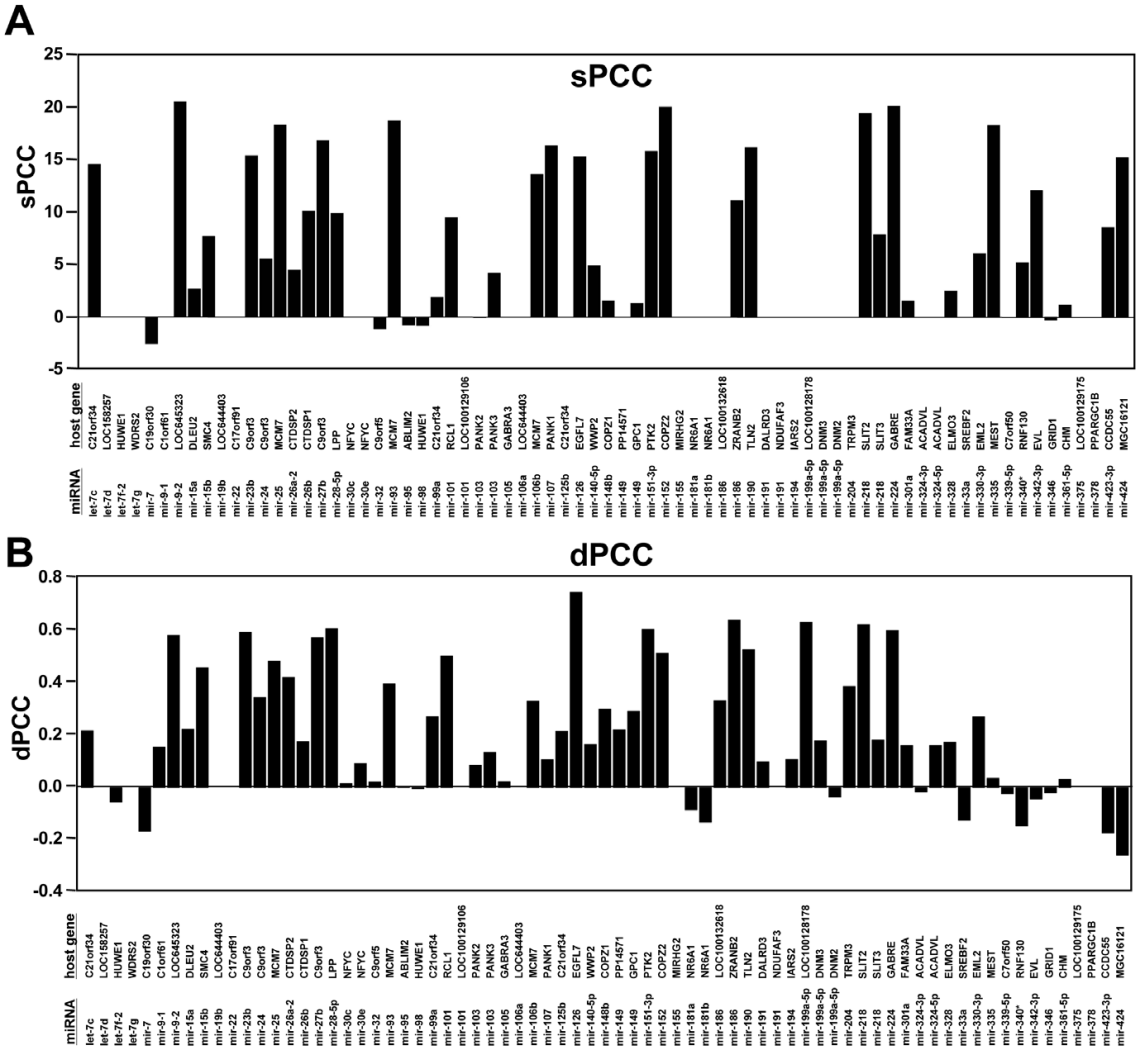
The endogenous miRNAs positively correlate with their host genes. (*A*) sPCC and (*B*) dPCCs values are given for the 73 miRNA/host gene pairs represented in the Q data set and gene expression data sets which occurred with at least one of the methods. Reanalysis of the data by comparing sPCC/30 with dPCC values with a cutoff of 0.2 revealed that the two methods do not differ in their ability to predict host genes (either by paired t-test or paired Wilcoxon test).

Many miRNAs are encoded within co-located genes (host genes) and share promoters with them. Expression of these miRNAs is driven by the promoters of their host genes, and positive correlations between expression of miRNAs and their host genes have been reported [22], [24]. By utilizing this information, we analyzed how often the co-transcription of miRNAs and their host genes could lead to positive correlations in the NCI60 data sets. Of the 136 miRNAs, 65 are encoded within host genes (Figure 2). In both the sPCC and dPCC analyses the number of positive correlations between host genes and their co-located miRNAs far out-numbered the ones of negative correlations (Figure 2A and 2B). In contrast the analysis with the rsPCC method resulted in a random distribution of positive and negative correlations (data not shown). The results of the sPCC/dPCC methods were comparable, suggesting that the reproducibility of NCI60 data sets was high and the sPCC method to identify correlations was as good as the dPCC method in the case of single host genes.

**Figure 3:**
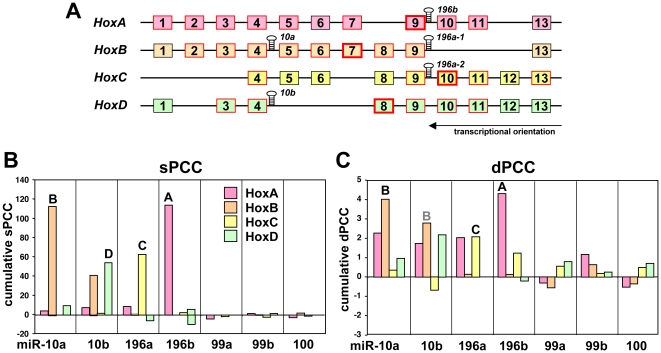
*HOX* genes positively correlate with the expression of the endogenous miRNAs that are encoded within these gene clusters. (*A*) Structure of the four mammalian *HOX* gene clusters with the location of the hosted miRNAs. *HOX* genes boxed in red were detected as positively correlated with the hosted miRNA using the sPCC method. For each cluster the *HOX* gene most strongly correlated with the miRNA that is in that cluster is boxed in bold red. (*B*) sPCCs of all individual *HOX* genes in each cluster were cumulated and plotted against the members of the *miR-10*/*miR-196* family and *miR-99a*, *-99b* and *-100*. (*C*) Same as *B* but generated using the dPCC method.

Next, we wanted to determine if the sPCC method would perform better than the dPCC method in identifying specific co-transcription. We took advantage of the *HOX* genes as a unique system of four gene clusters, each containing at least one intergenic miRNA. *HOX* genes regulate embryonic development and in mammals they are grouped into 4 clusters (*HOXA-D*) including 9 to 11 genes [22], [24]. Most of the HOX genes were positively correlated with co-localized miRNAs (red boxes in Figure 3A). Interestingly, the *HOXA*, *HOXC*, and *HOXD* clusters harbor one miRNA gene each and the *HOXB* contains two (Figure 3A). We first calculated dPCCs and sPCCs between the four miRNAs (*miR-10a*, *-10b*, *-196a*, *-196b*), which are encoded within the HOX clusters, and all human genes. We observed that in the sPCC method, in 3 of *4* of the clusters a HOX gene adjacent to the co-localized miRNAs had the highest positive correlation with these miRNAs out of ∼18,000 human genes (*miR-196b*/*HOXA9*, *miR-196a*/*HOXC10* and *miR-10b*/*HOXD8*; bold red boxes in Figure 3A). However, in the dPCC method, this was only true for two clusters (*miR-196b*/*HOXA10* and *miR-196a*/*HOXC10*) (data not shown). For each of the 136 miRNAs, we calculated sPCC values with genes in the 4 *HOX* gene clusters. The sPCCs of all *HOX* genes within a cluster were added up and the miRNAs were ranked according to the cumulative sPCC for each *HOX* cluster (Figure S2). Remarkably, for each cluster there was one miRNA that most clearly correlated with the expression of the *HOX* genes in that cluster (red column in Figure S2), and in each case it was the miRNA encoded within that cluster. To further compare the performance of the sPCC and dPCC methods we plotted the cumulative sPCC and dPCC scores for each *HOX* gene cluster versus the correlating miRNAs (Figure 3B and 3C). The cumulative sPCC and dPCC of the negatively correlating *HOX* genes are also shown but were negligible. We also included *miR-99a/99b* and *miR-100* in this analysis because they share extensive homology with *miR-10a* and *miR-10b* [25]. Once again, the sPCC performed better than the dPCC method detecting the correct *HOX* gene cluster for each correlating miRNA against a minimal background signal from other clusters.

In summary, these data demonstrate that the steady state system of the NCI60 mRNA and miRNA data is useful in detecting biologically meaningful connections between miRNAs and their host genes. The sPCC method, which was designed to mimic a miRNA titration experiment, was superior to the dPCC method in two assays (negative correlation with expressed mRNAs and *HOX* gene cluster correlation) used for characterizing our approach. The sPCC method was, therefore, used in the subsequent analyses.

### miRConnect.org, a searchable web interface to explore connections between miRNAs and their biological effector genes

As introduced above, in addition to actual target genes, genes that are not predicted to be miRNA targets as well as the large number of both positively and negatively correlating genes may hold important information with respect to the status of miRNA cellular expression levels, which may provide insight into the biological activities of miRNAs. All negatively and positively correlating genes for the 136 miRNA and the 25 miRNA families determined with both the dPCC and the sPCC method in the Q data set, as well as the information on how many of them are predicted targets by TargetScan 5.0, can be found under miRConnect-Q at a searchable web interface: miRConnect.org (or miRConnect.net)

### Clustering of miRNAs based on overlap of their correlating genes

Our data suggested that using the NCI60 data sets and the sPCC method could be useful to detect biologically relevant connections between miRNAs and their downstream effector genes, which might provide new insights into biological activities of miRNAs. We argued that genes either negatively or positively correlated with a specific miRNA will be equally important because each set may contain markers of a biological status regulated in opposite directions. For example, *E-cadherin* and *Vimentin*, which positively and negatively correlate with the expression of the *miR-200* family, respectively (*CDH1* and *VIM* in Table 1), both point at the EMT-related function of *miR-200*. Therefore, we suggested that either negative or positive correlations might independently define a specific biological status of a miRNA or a group of miRNAs.

**Table 1:** sPCCs for a number of known EMT marker genes.

Mesen. Gene	sPCC	Epith. Gene	sPCC
VIM	−15.95	CLDN7	22.83
TWIST	−15.13	CLDN4	19.77
SPARC	−13.29	KRT8	17.96
ZEB1	−13.05	JUP	17.94
CD44	−8.20	CDH1	17.20
SMAD4	−7.73	OCLN	15.53
FN1	−6.22	KRT18	14.82
ZEB2	−5.65	CTNND1	11.37
MMP2	−4.67	KRT14	10.38
CDH2	−4.63		
MMP14	−4.06		
MMP16	−3.91		
TGFB1	−3.34		
FGF2	−3.25		
CDH11	−2.51		
SMAD2	−2.51		
SNAI2	−2.33		
FGFR1	−1.89		
TNC	−1.45		
COL3A1	−1.23		

Only sPCCs of >1 and <−1 are shown.

**Figure 4:**
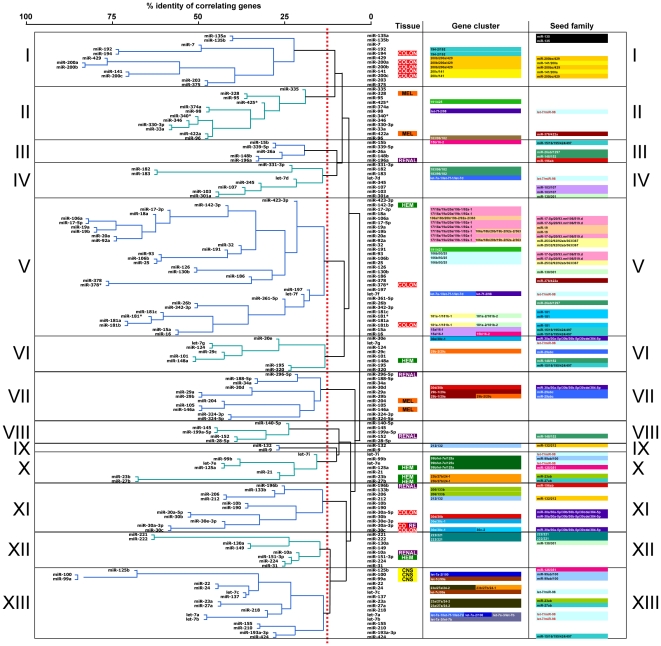
Cluster analysis of 136 miRNAs based on a pairwise comparison of positively correlating genes using the sPCC method. The miRNAs were divided into 13 functional clusters (I–XIII). Information is given for each miRNA on tissue specific expression, genomic co-localization, and seed family. Stippled line: threshold of 12.5% of groups that was chosen to defined the 13 clusters.

In order to test this assumption, we selected the top 2000 positive and top 2000 negative correlations and the corresponding genes for each of 136 miRNAs, and performed a hierarchical clustering to group the 136 miRNAs. We chose 2000 as a cutoff because this number covered about 10% of all genes, which should result in the elimination of most background noise. The clustering, which is based on pair-wise comparisons represents the intersection of the genes that significantly correlate in their expression with the expression of two different miRNAs (Figure 4 and Figure S3). When positively correlating genes were assessed, a number of miRNAs tightly clustered together (Figure 4). Similarly many of the same miRNAs clustered together when negatively correlating genes were used (Figure S3). Although numerous mechanisms may be responsible for this clustering, we will refer to these as “functional clusters”.

Interestingly, all 5 members of the *miR-200* family were tightly clustered, consistent with their similar biological activity (cluster I in Figure 4 and cluster VI in Figure S3). In addition to *miR-200*, a number of other structurally-related miRNAs formed functional clusters according to the shared numbers of their effector genes. Several seed families, such as *miR-181abc*, *miR-19ab*, *miR-221/222*, *miR-103/107* and *miR-135ab*, were clustered together tightly. In contrast, members of several other seed families were found scattered across different functional clusters (e.g. the *let-7* or the *miR-30* family). This phenomenon suggested that the grouping of miRNAs was partly based on, but not restricted to miRNA seed sequences.

The clustering of miRNAs in this analysis was partially due to the fact that some family members are part of the same transcriptional unit. miRNAs that share chromosomal co-localization and also found in the same functional clusters included the transcriptional units of *miR-106b/93/25*, *miR-17∼92*, *miR-194-2/192*, *miR-183∼182*, *miR-99b/let-7e/125a*, *miR-206/133b*.

In some cases, miRNA clustering was based on neither the seed match nor the genomic localization. For example, the members of both the *miR-141/200a* and *miR-200bc/429* seed families were clustered together although they comprise two gene clusters on separate chromosomes (cluster I, “Gene cluster” column in Figure 4).

The NCI60 cell lines represent 9 different human cancers. To determine whether the observed clustering of miRNAs was in part due to tissue specific expression of either miRNAs or mRNAs, we identified miRNAs that were preferentially expressed in any of the 9 human cancer types (Table S4; Table S5). Some correlations with tissue of origin were found. For example, both clusters I (including *miR-200* family and *miR-194*) and XI (including the *miR-30* family) contained most of the miRNAs that are enriched in colon cancer cells (Figure 4). Colon cancer cells may have a more epithelial-like characteristic than most other cancer cell lines.

In summary, these data suggested that while common seed sequences, chromosomal co-localization, and tissue specific expression likely affected the co-expression of miRNAs with certain genes and hence their grouping, many miRNAs were clustered for other reasons. A major factor that determines clustering could be the biological function of a miRNA, since the clustering is based on the intersection of gene sets that positively correlate significantly with two paired miRNAs. For example, *miR-200*, *-203*, *-375* and *-7*, which are grouped together in Figure 4 and Figure S3, do not have the same seed sequence, genomic colocalization or specific expression in same tissue. The reason for them to group together seems to be that they share similar biological function in EMT regulation.

### Identification of miRNA families involved in cell growth regulation

**Figure 5:**
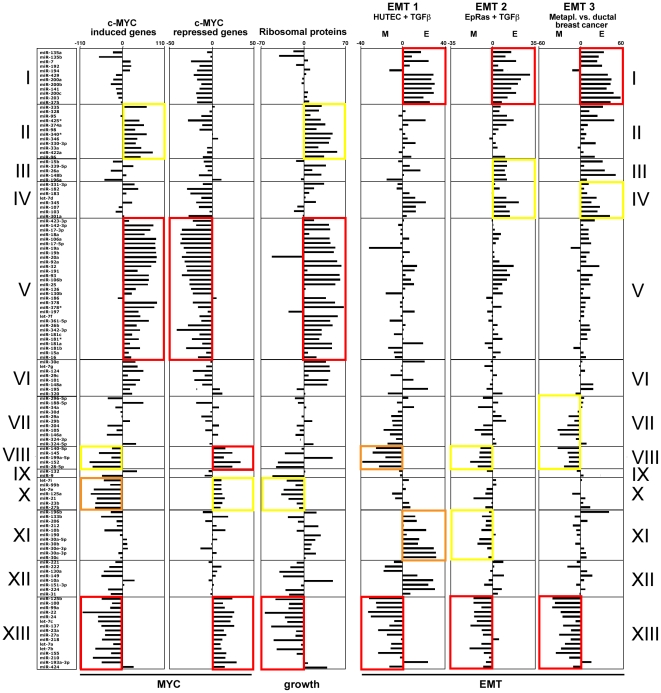
Correlation between selected gene signatures and the 13 miRNA functional clusters described in **Figure 4**. From left to right: Signatures of c-Myc regulated (induced and repressed) genes, ribosomal proteins (RPs) and 3 groups of EMT-related genes. For the c-Myc and RP signatures, each bar represents the number of genes that negatively correlate with a miRNA subtracted from the number of positively correlating genes. Details on the gene signatures and the calculation of the E and M factors used to analyze the EMT signatures are described in the Method section. Wilcoxon Rank-Sum Test was used to calculate significance for each functional cluster against the other clusters. Red, p<0.0001; orange, p<0.001; yellow, p<0.01.

*c-MYC* is not only a general regulator of miRNA function and expression, but also itself regulated by miRNAs [26], [27]. To determine whether grouping miRNAs according to the identity of correlating genes would detect the connection between miRNAs and *c-MYC*, we used lists of genes that are either upregulated (460 genes) or downregulated (211 genes) by *c-MYC* (obtained from http://www.myc-cancer-gene.org/) and determined how many of them were either positively or negatively correlated with the expression of each of the 136 miRNAs. The result is visualized in Figure 5. The significance of enrichment of correlating genes was determined by performing a Wilcoxon Rank-Sum Test. The significance level is indicated by boxes with different colors. Clearly, certain clusters of miRNAs were positively, and others were negatively correlated with either *c-MYC* induced or *c-MYC* repressed genes. Examples are the miRNAs in cluster V which positively correlated with *c-MYC* induced genes and negatively correlated with *c-MYC* repressed genes (Figure 5). In contrast almost all miRNAs in clusters VIII, X and XIII showed the opposite behavior.

*c-MYC* is known to be an important regulator of cancer cell growth in part by regulating ribosomal biogenesis [28]. To confirm the correlation found between clusters of miRNAs and the growth of cells, we identified all ribosomal protein genes (RPs, total number in the data set = 99) with a sPCC of either >1 or <−1 in the data sets of correlating genes for each individual miRNA. We would argue that a miRNA that is positively correlated with a large number of RPs suggests a strong activity in cell growth. In contrast miRNAs that are strongly negatively correlated with RPs would indicate growth suppressive activity. This was confirmed with the *c-MYC* regulated genes, in that every cluster of miRNAs that positively correlated with *c-MYC* induced genes also positively correlated with the expression of RPs, and every cluster that negatively correlated with the expression of *c-MYC* induced genes also negatively correlated with the expression of RPs (Figure 5). As the most significant examples, both *c-MYC* induced genes and RPs positively correlated with the miRNAs in cluster V, but negatively correlated in the miRNAs in cluster XIII (Figure 5). We therefore concluded that clustering miRNAs according to the expression of *c-MYC* regulated genes or RPs allowed us to assign growth promoting and growth suppressing activities to different miRNA functional clusters.

### Identification of epithelial and mesenchymal specific miRNAs

After we established the power of our analysis to functionally link miRNAs with mRNAs, we sought to test the data in identifying mRNAs that correlate with *miR-200* and known EMT markers. To identify EMT relevant genes that correlate with *miR-200*, we determined the total activity of all 5 *miR-200* family members in the NCI60 cells. A number of well known EMT marker genes and their sPCC are listed in Table 1. Canonical mesenchymal genes such as *Vimentin* or *Twist* gave highly negative sPCCs, and well-known epithelial marker genes such as *E-cadherin* or *cytokeratins* 8 and 18 produced highly positive sPCCs.

While all 5 *miR-200* family members were tightly clustered together regardless of the approach used for clustering (cluster I in Figure 4 and cluster VI in Figure S3), some other miRNAs were co-clustered with the *miR-200* family, such as *miR-7*, *miR-203*, *miR-375*, *miR-192* and *miR-194*. In order to determine whether these miRNAs were positively correlated with the enrichment of epithelial genes and negatively correlated with mesenchymal genes, we analyzed three EMT-related gene signatures, each representing a particular type of EMT process. EMT signature 1 was derived from primary human tubular epithelial cells (HUTEC) before and after a 4-day treatment with *TGF-β* [29]. EMT signature 2 stemmed from Ha-Ras-transformed EpH4 cells before and after *TGF-β* induced EMT [30]. EMT signature 3 was derived by comparing the differences in gene expression of metaplastic carcinomas of breast (MCBs) and ductal carcinomas of breast (DCBs) [31]. The three EMT gene signatures therefore represented cases of a normal tissue, a transformed cancer model and a primary human cancer, respectively.

In all three models, all 5 members of the *miR-200* family segregated in the cluster including the most epithelial miRNAs together with *miR-7*, *miR-203*, and *miR-375* (cluster I in Figure 5). Figure S4A shows all miRNAs sorted from highest to lowest by their E/M nature. For this analysis a universal EMT signature was generated by taking the average of the EMT signatures 1–3. This universal EMT signature was compared to miRNA regulated genes determined using the sPCC method. Interestingly, miRNAs identified as having the most mesenchymal nature, such as *miR-100*, *miR-125b* and *miR-99a*, were also clustered together in all three EMT signatures (cluster XIII in Figure 5). Figure S4B shows the actual similarity of positively correlated genes among the miRNAs with the highest and lowest E/M nature (colored columns), generated by calculating overlapping percentages in positively correlated genes between 16 miRNAs and the total activity of the entire *miR-200* family. The percentages among the *miR-200* family members ranged from 72% (for *miR-141*) to more than 81% (for *miR-200c*). The *miR-200* miRNAs were closely followed by *miR-203* (55%), *miR-7* (48%) and *miR-375* (45%). miR-425* showed a much lower degree of identity in correlating genes (22%). However, for the 7 most mesenchymal miRNAs, virtually no genes were found to overlap with the genes that correlated with expression of *miR-200* (blue columns in Figure S4). These results further validated the NCI60 data set as well as the sPCC method, and identified novel, putative EMT-regulating miRNAs.

**Figure 6:**
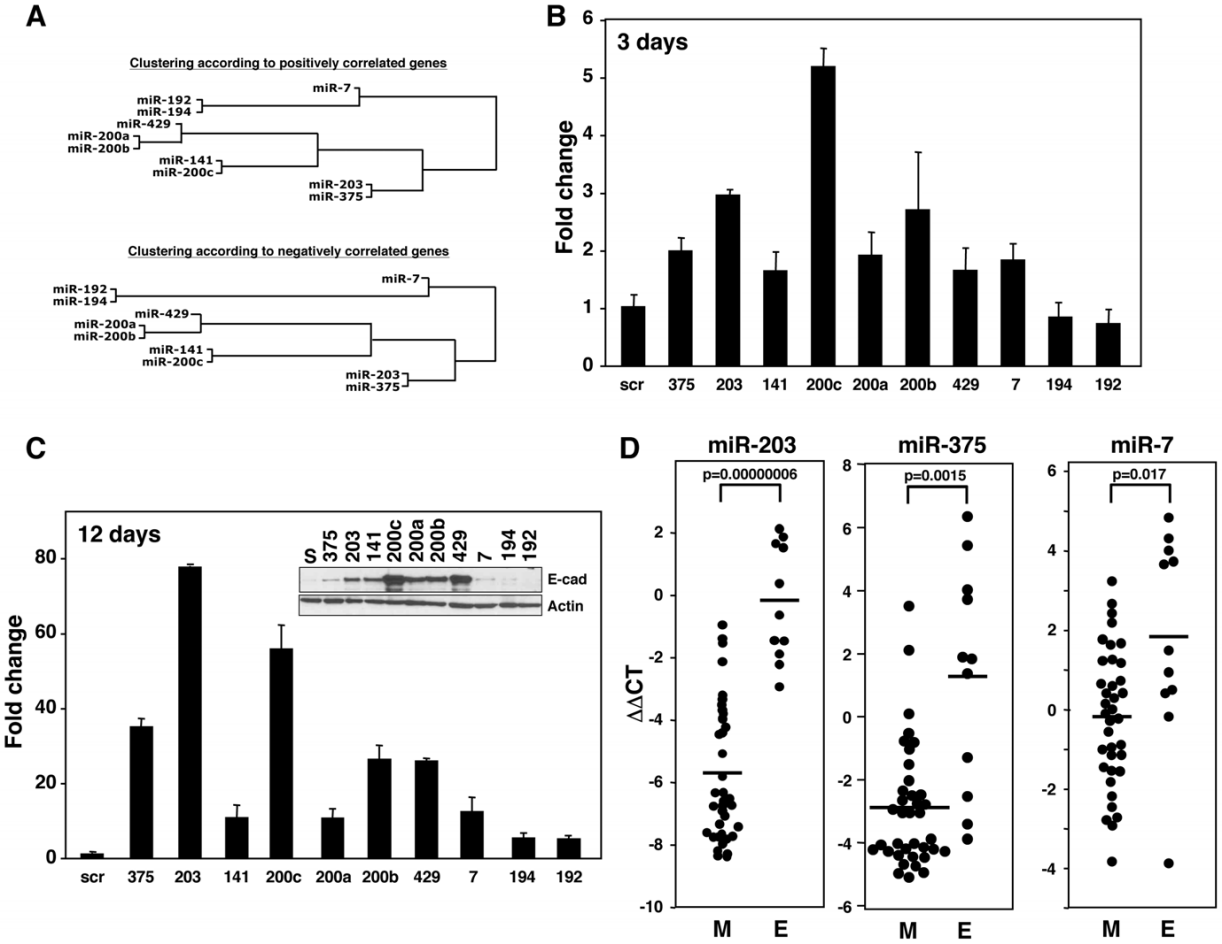
Validation of three predicted EMT-regulating miRNAs. (*A*) The EMT-related miRNA subcluster derived by using either positive (upper) or negative (lower) sPCC values as described in Figures 4 and S3. (*B* and *C*) Ability of miRNAs in the epithelial cluster to cause upregulation of *E-cadherin* in mesenchymal ACHN cells. ACHN cells were either transfected (*B*) once or (*C*) 3 times with the indicated miRNAs, and after (*B*) 3 days and (*C*) 12 days respectively, the expression of *E-cadherin* mRNA was quantified by real-time PCR. The Western blot inserted in *C* confirmed that the upregulation of *E-cadherin* mRNA also resulted in a induction of *E-cadherin* protein. (*D*) Comparison of the expression of the three new EMT-regulating miRNAs between the epithelial (E) and mesenchymal (M) cell lines among the NCI60 cells recently described [11].

There were 10 miRNAs which were consistently grouped in an “epithelial” cluster, regardless of whether they were clustered based on the positively or negatively correlated genes (Figure 6A). Consistent with the analysis shown in Figure 5 and Figure S4, *miR-203*, *miR-7* and *miR-375* were more closely related to miR-200 than miR-192 and miR-194. This was also confirmed by a Principal Component Analysis for 136 miRNAs based also on positively correlated genes (Figure S5). *miR-135a* and *miR-135b* were excluded from this analysis based on a previous analysis when only the sPCC values were used to produce the cluster map rather than the degree of overall in coexpressing genes (data not shown). In addition, these two miRNAs also did not cocluster with the other epithelial miRNAs in the Principal Component analysis (Figure S5). All the cluster I miRNAs were found to co-cluster except for the two *miR-135* miRNAs. While *miR-135a* and *miR-135b* were above the chosen cutoff in the analysis shown in Figure 4, they are the most remotely related miRNAs to the core of the cluster I miRNAs. To test whether the miRNAs in the epithelial cluster were merely markers or were actively involved in the maintenance of epithelial cells, we transfected each of the 10 miRNAs in this cluster (Figure 6A) into the mesenchymal cell line ACHN, which does not express *E-cadherin* [11]. Transfection of *miR-7*, *miR-203*, or *miR-375* in addition to the members of the *miR-200* family caused upregulation of *E-cadherin* mRNA within 3 days (Figure 6B). After three transfections over 12 days, the level of *E-cadherin* mRNA induced by *miR-203* was even higher than that observed with *miR-200c* (Figure 6C). However, on the protein level, *miR-203* was less effective than *miR-200c*, but still significantly effective in upregulating E-cadherin (Figure 6C). This analysis identified *miR-7*, *miR-203*, and *miR-375* as novel EMT regulators in cancer cells and validated the core part of the cluster I miRNAs (Figure 4) as regulators of EMT. Consistently, *miR-7*, *miR-203*, and *miR-375*, closely aligned with the *miR-200* family, were preferentially expressed in the epithelial cell lines that we have recently described among the NCI60 cells (Figure 6D) [11]. This suggests their epithelial role *in vivo*.

The identification of *miR-7*, *miR-203*, and *miR-375* as markers for epithelial cancer cells validated our hypothesis that the clustering of miRNAs according to correlating gene overlap reveals new functional similarities between miRNAs. We concluded, therefore, that this analysis grouped miRNAs primarily according to their function rather than their predicted targets, seed families or genomic organization. Thus, miRConnect-Q can be used to identify novel miRNA targets and downstream biological effectors.

### Expanding miRConnect: miRConnect-L

miRConnect-Q is based on a set of expression levels of 208 miRNAs quantified by real-time PCR. Recently an additional set of miRNA expression data became available based on LNA-oligonucleotide arrays (the “L” data set) [32]. This new data set contained information on 571 individual miRNAs for which the oligonucleotides were uniquely selective. This number of miRNAs allowed us to generate data on 53 miRNA seed families. We again calculated the expression of the miR-200abc/141/429 custom family. We generated correlation data sets using either the sPCC or the dPCC method (with some modifications - see Method section). Similar to the Q-data set we performed the analysis on the correlation of miRNAs with their host genes (see Figure 2) and the expression of the miR-10ab/196ab families with Hox genes (see Figure 3) and again found a similar level of positive correlations between the miRNAs and the host genes (data not shown). Similarly both the analysis of EMT genes (see Table 1) and the cluster analysis (see Figure 4) gave confidence that the new L data were as useful as the Q data (data not shown). Both data sets now cover a total of 583 individual miRNAs/376 miRNA seed sequences and 54 miRNA seed families with more than one family member. All data can be found as miRConnect-Q and miRConnect-L at miRConnect.org.

## Discussion

While the ability to predict miRNA targets represented a great advancement in the field and has helped to identify many miRNA targets, the false positive rate of all of the algorithms is high. This hampers accurate prediction of miRNA function based solely on the list of predicted targets. While it is well established that the co-expression of miRNAs which are part of the same transcriptional unit is often indicative of a common biological function, it has not been consistently so, and also has not permitted accurate grouping of miRNAs according to their function. We now present a new method to group miRNAs, which relies on identification of co-expressed effector genes for which a large amount of functional data is available.

Our analysis is based on three premises: 1) Cancer-relevant miRNAs act in pathways that regulate cellular differentiation stages which can be recognized by the signature of expression of a number of stage-specific marker genes (i.e. *E-cadherin* for epithelial cells or early carcinomas). 2) A substantial change in the expression of these marker genes is brought about by a relatively small change in the expression of genes that are actual miRNA targets (i.e. *ZEB1/2*). MiRNAs therefore are part of amplification loops. 3) A cancer-relevant differentiation stage is characterized as much by expression of positively correlating marker genes (i.e. *E-cadherin*) as by negatively correlating ones (i.e. *Vimentin*) which are not direct targets of the correlating miRNA (i.e. *miR-200* does not target *Vimentin*). Our method is complementary to existing methods and, in conjunction with prediction algorithms and improved methods for confirmation of actual *in vivo* targeting events [33], should allow better prediction of the cancer relevant function of miRNAs.

A number of studies have addressed the question of miRNA function using biocomputational approaches. These approaches have all used target predicting information based on sequence complementarities (e.g. TargetScan).

In one early work, a computational strategy to predict miRNA regulatory modules (MRMs) was developed using bipartite graphs to model miRNA-mRNA binding structures [34]. An extension of the MRM concept was incorporated into a later computational method to discover functional miRNA-mRNA regulatory modules (FMRMs) [35]. This method associated expression data and specific biological conditions (cancer versus normal tissue) with the previous bipartite graph structure. An algorithm of population-based probabilistic [36] and an alternative method of rule-based learning [37] were also employed. However, these approaches all relied on known target prediction algorithms and/or gene ontology (GO) data, and were either theoretical, or did not consider actual expression data, or were based on rules that were generated using over-expression systems.

Two approaches [38], [39] used Bayesian Network methods to explore interactions between miRNAs and mRNA transcripts. The first report developed a probabilistic graphical model for miRNA regulation and variational learning for detecting miRNA targets. The second report developed a strategy of splitting and averaging of Bayesian networks (SA-BNs). In this method, miRNA-mRNA interactions in the NCI60 cell lines were split into different sample categories, and both up- and down-regulation information was included. These improvements were believed to capture stronger interactions than were detected using conventional Bayesian Networks. However, this study only described a subset of miRNAs and genes regulating EMT. The analysis culminated in predictions on signaling networks regulated by miRNAs using the Ingenuity pathway analysis software, which is based on published data and requires constant updating. While a Bayesian Network provides a powerful method for constructing regulatory networks, it has its limitations in predicting miRNA regulatory modules. 1) According to the definition of a Bayesian Network, loop structure is not allowed, which means a Bayesian Network cannot be applied to regulatory feedback loops as they are common between miRNAs and their targets (e.g. the relation between *ZEB1* and the *miR-200* family). 2) The learning procedure of a Bayesian Network is computationally exhaustive [40], and the space of possible structures is limited by gene number, as linear increases in gene number cause exponential growth of the required space.

A recent approach was based on the statistical enrichment of miRNA targeting signatures in annotated gene sets. It allowed prediction of a number of novel targets for various miRNAs and prediction of disease relevant pathways [41]. However, this analysis was again based solely on predicted functions of genes and predicted seed matches, and actual expression data were not considered. In addition, it used target prediction algorithms as well as GO data. The analysis was based on a number of assumptions and requires frequent updating, which may affect some of the reported findings. In contrast to the previous approaches, our study is based on actual and unperturbed gene expression data, and none of our key findings rely on any known target prediction algorithm or GO data.

### The in silico titration method sPCC

Due to the nature of how miRNAs function, we designed the sPCC method as an *in silico* titration. We argued that, in order to detect mRNAs whose expression was repressed by miRNAs, the miRNA had to be expressed. We predicted that very high expression of miRNAs would suppress expression of targets representing certain biological processes. In contrast, in cells completely lacking expression of miRNAs, targeted genes would either be expressed or not expressed as a consequence of other regulatory mechanisms. In other words the “pressure” of a miRNA on a gene should be best detectable by comparing cells with high versus low expression of a miRNA. In order to reflect this pressure, different weights were assigned to each of the cell lines based on their miRNA expression levels. One could argue that the classical PCC method might also be modified in this way. However, in practical terms, it would be arbitrary to add weights directly to each cell line. First, the PCC calculation is not a linear process, and hence any change within the PCC function may have uncontrollable consequences. Second, it would not be clear how much weight to assign to each cell line. To solve this problem, we adopted the “titration” method sPCC, with a gradient weighting approach for cell lines sorted according to miRNA expression. We decided to pick 30 cell lines as a threshold, for the following reasons: 1) the model adds weight at the level of cell line selection, so it is linear and additive for PCCs with different cell line selections (e.g. from pattern 30 to pattern 59); 2) the model fully considers the biological importance of the 30 cell lines with the highest expression of each miRNA, as every pattern includes them; and 3) the minimal number of 30 cell lines ensured the methodological stability, as PCC is highly unstable and easy to be interfered by background noise when its sample size is too small (e.g. <10). We intentionally reduced the importance of the rest of the 29 cell lines, rather than simply neglecting them. If we had performed the dPCC analysis by only using the 30 highest expressing cell lines, it would be problematic since different sets of cell lines would be selected for each miRNA. In order to test the stability of our threshold selection, we used a randomized sPCC (rsPCC) method as an internal negative control (Figure 1).

The performance advantage of the sPCC was obvious when the methods were tested for the ability to detect the conserved miRNA targets with the highest total TargetScan context score in the human genome. Only with the sPCC method the correlation increased with the more strongly predicted targets (Figure 1B). We chose to use the TargetScan predicted genes as opposed to a list of experimentally validated targets because TargetScan provides total context scores, which can be used to rank predicted miRNA-gene pairs (e.g. from top 50 to top 500). While most of these targets are not validated it has been demonstrated that the number of likely targets is greater towards the very top of each ranked list of targets [15]. Our sPCC analysis confirmed this. Furthermore, the sPCC method performed better in two other analyses (host gene and *HOX* gene cluster gene correlations) when compared to the dPCC method. Interestingly, only the sPCC was good enough to group miRNAs for exploring their potential biological functions. Regardless of whether positively or negatively correlating genes were used, the dPCC method failed to group some of the most biologically relevant miRNAs, such as members of the *miR-200* and *miR-17* families (data not shown). As an important control the sPCC method performed far better than the randomized rsPCC method. Hence, this method is robust enough to explore biological functions of miRNAs. We decided to let the user decide whether to use the dPCC or sPCC analysis on miRConnect.org. In summary, our data demonstrate that across all NCI60 cells the sPCC method performed best at both the theoretical and biological level, permitting detection of general connections between miRNAs and their downstream effectors.

### Host genes and *HOX* genes

One mechanism that determines co-expression of miRNAs and mRNAs is when both are driven by the same promoter. This is the case for miRNAs that are part of host genes. A similar situation occurs at the 4 human *HOX* gene clusters, which provided us with a unique system in which each cluster of multiple genes contained at least one miRNA gene. For both the host gene and the *HOX* gene cluster case we found a highly significant correlation between the expression of the gene(s) and the miRNAs that are linked to their co-transcription. Of the 73 host gene/miRNA pairs in our analysis of the Q data set, 37 showed a positive sPCC, but negative sPCCs were only found in 5 cases and they were of low significance (Figure 2A). The correlation of miRNAs with their hosting *HOX* genes was even more impressive. In each case a substantial number of *HOX* genes positively correlated with the expression of the miRNA in that cluster (9/11 *HOXA* genes with *miR-196b*, 8/10 *HOXB* genes with *miR-10a*, 5/9 *HOXC* genes with *miR-196a*, and 6/9 *HOXD* genes with *miR-10b*), and few *HOX* genes negatively correlated with these miRNAs. This analysis in particular illustrates the strength of the expression correlation that can be found using the NCI60 data analysis. The sPCC method achieved the highest positive correlations between the four miRNAs and their adjacent *HOX* genes. Out of ∼18,000 human genes the following HOX genes had the highest positive sPCC for the miRNAs that are coded within a HOX gene cluster: *HOXA9 and miR-196b*, *HOXB7 and miR-10a*, *HOXC10 and miR-196a* and HOXD8 and *miR-10b*. Interestingly, our data allow us to predict that *miR-196a-1*, which is part of the *HOXB* gene cluster, is not significantly expressed in cancer cells, since neither of the two methods of analysis detected any *HOXB* genes that were co-expressed with this miRNA (Figure 3B and 3C).

### Novel and unusual predictions that come out of our analysis

Our analysis makes a number of predictions that cannot be explained based on current knowledge. These include the prediction that *let-7* is a family of at least 6 different potential activities in cancer cells (functional clusters II, IV, V, VI, X and XIII in Figure 4). Our data now provide the impetus to look into this phenomenon. We hypothesize that nine different *let-7* family members do not just exist to allow tissue-specific expression of *let-7*.

The finding that seems to be most at odds with published data is our results on *miR-21*. *miR-21* is the miRNA upregulated in the most human cancers when compared to normal tissue [42], and *miR-21* was recently shown to induce neoplastic transformation when expressed as a transgene in mice [43]. Confirming published data, *miR-21* expression negatively correlated with two of its main validated targets, *PDCD4* [44], [45] (sPCC = −4.7) and *PTEN* [46] (sPCC = −1.05). *miR-21* has, therefore, been viewed as an oncogenic miRNA, and one would expect it to be one of the most growth-promoting miRNAs. It was surprising, therefore, that in our study *miR-21* was co-clustered with miRNAs that had a strong negative correlation with genes induced by *c-MYC* (functional cluster X in Figure 5), which would indicate a growth suppressing activity. We also determined the extent of correlation between the expression of miRNAs and 99 ribosomal genes as markers for cell growth. Interestingly and consistently with the results on the *c-MYC* regulated genes, expression of *miR-21* had a strong negative correlation with the expression of almost all ribosomal genes. In fact when the miRNAs were ranked according to the correlation with ribosomal genes, *miR-21* was the third most clearly negatively correlating miRNA (Figure S6). How could this finding be reconciled with the existing paradigm of *miR-21* being such a significant marker for cancer cells? We recognize that all reports identifying *miR-21* as being overexpressed in cancer cells were based on comparisons of cancer with normal cells [42], while our analysis does not include normal cells. Our finding may, therefore, have revealed a cellular function of *miR-21* that is not cancer specific. Interestingly, there is very little information on the physiological function of *miR-21* outside of cancer. However, two pieces of data provide a clue as to the physiological activity of *miR-21*. In the context of a cancer cell line, in an unbiased approach 94 different endogenous miRNAs were individually inhibited in HeLa cells. Inhibition of most miRNAs resulted in reduced growth of the cells. Only two miRNAs were identified as growth repressors, as their inhibition accelerated the growth of the cells. By far the most growth suppressing miRNA was *miR-21* [47]. In normal tissues *miR-21* has also been recognized as a paradoxical miRNA [48]. In the context of heart hypertrophy, *miR-21* was reported to inhibit hypertrophy of cardiomyocytes [49]. Based on these data and our analysis we predict that one of the physiological activities of *miR-21* is to inhibit, rather than to promote, cell growth.

### EMT markers and new EMT regulators

Induction of EMT induces changes in expression of thousands of genes in the human genome. Many of these changes involve sharp on-off regulation of genes. Examples of genes that are regulated in a switch-like manner are *E-cadherin* and *Vimentin* where the difference in the ratio of their expression across the NCI60 cells spans 8 orders of magnitude [11]. Neither *E-cadherin* nor *Vimentin* are direct targets of miRNAs that regulate EMT, but they are powerful markers for the two cellular stages of epithelial and mesenchymal cells. Interestingly, the remarkable similarity in the genetic programs of EMT is documented by the three EMT gene signatures used in this study, and the resulting similarity in correlation of gene expression with that of certain groups of miRNAs. Our analysis allowed us to identify and validate three new regulators of epithelial cells, *miR-7*, *miR-203* and *miR-375*. *miR-7* was previously shown to target the epidermal growth factor receptor (EGFR), a known regulator of EMT [50], and EGFR was also the gene with the highest TargetScan total context score that was most negatively correlated with *miR-7* expression (sPCC = −2.26; see miRConnect.org). *miR-203* was recently linked to EMT regulation and stem cells [51]. In our analysis, *miR-203* was a potent inducer of *E-cadherin* upregulation. Predicted targets include the EMT regulator Snail2 (Slug) which also had a low negative sPCC of −8.01 (miRConnect.org). Finally *miR-375* was recently reported to target 3-phosphoinositide-dependent protein kinase-1 (PDK1) [52] which promotes invasion and activation of matrix metalloproteinases [53]. We also identified a number of miRNAs that are co-expressed with mesenchymal genes. Among these we found *miR-155*, which we had already identified as most highly expressed in mesenchymal cancer cells [11], and this was recently confirmed by others [39].

### miRConnect-Q and miRConnect-L

On the miRConnect.org site we have made data sets available based on two different miRNA expression data sets. In a recent analysis the level of concordance between 4 different miRNA expression data sets obtained with the NCI60 cells was moderate [32]. The PCC between the L and the Q data sets used in our study was found to be 0.56. Nevertheless we found that the discrepancies were often in miRNA species with relatively low expression levels. Data on major miRNA families including *let-7*, *miR-200* or the *miR-17∼93* family were more consistent (data not shown). Regardless of the nature of the differences they must not necessarily be due to fluctuation between the analyses. The two platforms that generated the Q and the L data sets are very different and each has both advantages and caveats. The LNA-based arrays that underlie the L data set cannot discriminate between mature miRNAs and their pre-miRs but are more robust in detecting different isomiRs. In contrast, the Q data set relies on real-time PCR involving loop primers. While they are highly selective for the mature miRNAs they cannot distinguish between certain isomiRs. This could be relevant because in a recent study it was demonstrated that miRNAs exit as multiple isomiRs and this varies among species and tissues [54]. We therefore decided to make the analysis based on both data sets available on miRConnect.org.

Expression levels of miRNAs and mRNAs have been correlated previously in attempts to establish causality [22], [55], [56], [57], [58]. This has been complicated by the fact that certain mRNAs are both regulators of miRNA expression/biogenesis and targets of these miRNAs. Examples are *c-MYC* and *let-7*, and *ZEB1*/*ZEB2* and *miR-200* [59], [60]. Our work provides an additional way to link miRNA expression to biological function (i.e. *miR-200* to EMT or *miR-10/miR-196* to *HOX* gene expression) without the need to validate specific mRNAs as miRNA targets.

Based on our data, we propose to focus more on individual pathways when assigning cancer relevant functions to miRNAs and less on global activities. We believe that the functional grouping of miRNAs using miRConnect will significantly aid miRNA researchers and will provide a novel resource to the field that complements the study of miRNA function based on their seed sequences. We propose to group miRNAs families according to seed families, gene clusters, and functional correlations.

## Methods

### The miRNA data sets

#### The Q data set

The real time PCR data (ct values) for 207 individual miRNAs was previously described [20], and expression data for each miRNA can be found at http://dtp.nci.nih.gov/mtargets/download.html as WEB_DATA_ISRAEL_MIR.ZIP. For each miRNA the number of cell lines with an expression level above the limit of detection was determined. The real time PCR data set for *miR-429*, which was not part of the original data set, was provided by Dr. Arti Gaur (Dartmouth Medical School, Lebanon, NH). Expression of the miRNA was determined by normalizing the data set as previously described [11]. Each miRNA was assigned to a miRNA seed family (miR_family) as defined by TargetScan.org. To develop the sPCC method, the integration of miRNA IDs and measurements was done using a customized Java platform (Netbeans java console project MicroRNAparser). A few miRNAs were not explicitly included in the TargetScan family assignments and a few used different nomenclatures than that used in TargetScan. In these instances miR_family assignments were made based on sequence (see Table S5). For any miR_family that contained two or more miRNAs detectable in at least 30 cell lines, an average expression of 59 cell lines (family_average) was calculated for the mir_family. The miRNA and miR_family data were used to generate linked subsets of cell line data. Each member of these subsets was used as a seed for COMPARE analysis, and the sets of COMPARE results, linked by miRNA or miR_family, were aggregated.

#### The L data set

The LNA array based expression data (log2 ratios) of 955 individual miRNAs were recently described [32] and can be found at GEO (accession number: GSE26375). Of these 955 miRNA probes 571 were specific for only one miRNA (with no crossreactivity with other miRNAs) and were therefore used in our study. Total expression of an entire seed family was determined by adding the individual expression values of all members. To have more control of the analysis locally we chose to analyze the L data set by using R software.

### The mRNA data sets

The analyses performed in this work covered a total of 130,368 individual gene array probes in the MICROARRAY_ALL target set available at the DTP site (http://dtp.nci.nih.gov/mtweb/search.jsp). Of these, 82,564 gene probes had GeneCard identifiers (distributed over 4 array platforms: STANFORD: 7328 genes, GENELOGIC_U95: 34,309 genes, GENELOGIC_U133: 31,266 genes, and NOVARTIS: 9661 genes) and they represented 18,462 uniquely known genes in the human genome.

### COMPARE analysis

COMPARE analysis was used to generate correlations between the data in the miRNA Q data set and the gene array data. COMPARE is a computational tool available through the NCI DTP web site. (http://dtp.nci.nih.gov/compare). It was developed in the early 1990's to explore the results from the NCI60-cell line screen [23] but has since been adapted as a web based tool [61], [62]. The COMPARE algorithm ranks the members of the data set based on the similarity of the responses or expression levels of a single set of cell line measurements. The similarity metric used is a Pearson Correlation Coefficient (PCC).

### The dPCC method

To generate miRConnect-Q, each of the 136 miRNAs with at least 30 cell lines with detectable expression and each miRNA of the 25 families was used as a seed for a single COMPARE analysis against 18,747 genes. The microarray features for all COMPARE correlations were collected. Correlations for microarray features associated with named genes (GeneCard/Human Genome Organisation/HUGO, www.HUGO-international.org) were then determined by averaging the individual correlations for each gene. To generate miRConnect-L, the expression profiles of 571 miRNAs/53 miRNA families and 18,747 genes in 59 cell lines were used to calculate a Pearson Correlation Coefficient (PCC) between each miRNA and each gene. Then, the average values of PCCs between each miRNA and each gene were calculated across all 4 gene array data sets for gene expression to normalize for variation among different microarray platforms. These averaged values were designated direct PCC (dPCC). During this process missing values were excluded. The top 2000 positive and negative dPCCs (covering about 10% of all genes) were collected to perform the EMT, *c-MYC*, and RPs correlations. This resulted in the elimination of 97% of the correlations with dPCCs corresponding to values between −0.3 and +0.3.

### The sPCC method

To generate miRConnect-Q, detectable miRNAs and miRNA families were used to generate linked subsets of cell line expression data, referred to as “patterns”. Before creating the patterns, the cell lines were ranked by their miRNA expression from highest to lowest (or decreasing average expression in the case of miRNA families). For each miRNA or miRNA family, pattern 30 consisted of the first 30 cell lines (those with the highest levels of expression), pattern 31 included all of the cell lines from pattern30 and the cell line with the next highest level of expression, pattern 32 included all of the cell lines from pattern 31 and the cell line with the next highest level of expression, and so on. The last pattern, pattern 59, consisted of all of the cell lines and completed the set of linked patterns each with 30 members. Each individual pattern was used as a seed for a single COMPARE analysis against the MICROARRAY_ALL target set. After COMPARE had been run on all 30 linked patterns, the sum of the COMPARE correlations (sum_correlation) for each microarray feature for the 30 patterns was determined. Correlations for microarray features associated with named genes (GeneCard/HUGO) were then determined by averaging the individual correlations for each gene. The top 2000 positive and negative sPCCs (covering about 10% of all genes) were collected to perform the EMT, *c-MYC*, and RPs calculations. For miRConnect-L, R software was used to generate sPCC values. Subsequently the sum of PCCs for these 30 patterns was added up to calculate each sPCC value. Variations within sPCCs across different gene array platforms were eliminated by taking average values across different platforms.

### The rsPCC method

The only difference between the rsPCC method and the sPCC method was that rsPCC generated patterns according to randomized sorting of cell lines. Each rsPCC was repeated 10 times, and average of the calculations was taken.

### Processing TargetScan

Predictions and underlying data for TargetScan version 5 were downloaded at http://www.targetscan.org. The TargetScan data sets were used to generate predictions for microRNA families, to limit those predictions to conserved miRNA, and to generate custom predictions for families that we defined. These manipulations were based on the descriptions of the TargetScan algorithms which were included on the TargetScan 4.2 web site, with slight modifications to accommodate changes in the downloadable data as follows: Predictions were limited to those predictions that included at least one human example, context_scores >0 were not included in the determination of total_context_score, and the aggregate prediction for a miRNA family was the minimum total_context_score for any miRNA that was a member of that family.

### Generation of the searchable web interface miRConnect

A table of miRNA/mRNA pairs was loaded into a (MySQL) database along with their correlation values, mRNA chromosome locations, and function annotations (downloaded from the HUGO). A web interface was built to filter and extract data from this table. Genes can be shown that correlate positively or negatively with a given miRNA or miRNA family, according to the dPCC or the sPCC method. If no miRNA is selected but instead a list of mRNA gene IDs are entered, then miRNAs that correlate will be listed. For negative correlations, TargetScan total_context_score was also provided. Functional annotation search words can be used to constrain the output, and all output tables are downloadable in spreadsheet-ready form. The site contains all data on miRConnect-Q and -L and is accessible at miRConnect.org.

### Host gene and *HOX* gene analyses

The miRNA host genes were retrieved by comparing the genomic positions of miRNAs and genes as recently described [63]. All the analyses were based on the genomic coordinates of human assembly build 36.1. For both miRNA/gene association methods, we obtained dPCC/sPCC statistics between host genes or *HOX* family genes and the miRNAs. In order to spotlight the miRNAs associated with *HOX* gene clusters, for each miRNA we summed up the dPCC/sPCC for all the members in each *HOX* gene clusters.

### Identification of cancer specific miRNAs

Specifically expressed miRNAs in each type of cancer were screened out by Significance Analysis of Microarrays (SAM) with a manually adjusted False Discovery Rate (FDR). FDR was set as 0.01 (Table S4).

### Hierarchical clustering of miRNAs and Principal Component Analysis based on identity of correlating genes

For the sPCC positively and negatively associated gene lists, we queried all possible pairs of miRNAs with either the top 2000 sPCC values (for positive correlations) or bottom 2000 sPCC values (for negative ones) and calculated the percent identity of overlapping correlated genes between any two miRNAs. Thus 136×136 matrices for the overlapping gene numbers were generated. Then both matrices were transformed to distance matrices by using Euclidean distance measure (R function: as.dist). Complete linkage method was used to find similar clusters (R function: hclust) for dendrogram plotting. Functional clusters of miRNAs were defined by miRNAs that shared more than 12.5% correlating genes (R function: cutree). Principal component analysis (PCA) was adopted to do a orthogonal transformation, converting correlated miRNAs into an equal size of uncorrelated variables called Principle Components (PCs). The 136×136 matrix for the positively overlapping gene numbers was used to do orthogonal transformation using the R function prcomp. The two principal components with the highest score were used to generate a PCA plot.

### The gene expression signatures

Two sets of gene signatures were used. 1) *c-MYC* regulated genes. A non-redundant list of 460 genes upregulated by *c-MYC* and 211 genes suppressed by *c-MYC* was obtained from the *c-MYC* target gene database (http://www.myccancergene.org/) [64]. 2) Genes regulated during EMT. Three EMT gene signatures from previous studies were used in the analysis. There are 86, 72, and 121 epithelial genes and 59, 79, and 87 mesenchymal genes in the studies of Campanaro et al. (EMT 1), Jechlinger et al. (EMT 2), and Lien et al. (EMT 3) [29], [30], [31], respectively. We determined whether the expression of every gene in these lists negatively or positively correlated with the expression of the 136 miRNAs. The number of genes whose expression negatively correlated with a given miRNA was subtracted from the number of genes that positively correlated and plotted across all 136 miRNAs in the cluster analysis using either the dPCC or the sPCC method. Statistically significant positive correlations between gene expression and the expression of miRNAs in each of the 13 clusters detected with the sPCC method were determined using the Wilcoxon Rank-Sum Test. A p value of 0.01 was considered significant. For the EMT analysis an epithelial/mesenchymal (E/M) factor was determined as follows: (negatively correlating M genes - positively correlating M genes)+(positively correlating E genes - negatively correlating E genes) = E/M factor. The E/M factor was used to rank miRNAs according to their E/M nature in Figure S4.

### Testing epithelial specific miRNAs for their ability to upregulate *E-cadherin* in mesenchymal cells

Mesenchymal ACHN cells which express little *E-cadherin* were serially transfected with pre-miRNAs (Ambion) up to 3 times as described [11]. Western blot analysis and real time PCR for *E-cadherin* were performed as described [11].

### Statistical analyses

R statistical program v2.10 (http://www.r-project.org/) was used to perform microarray data manipulation, dPCC calculations, sPCC/dPCC/rsPCC comparisons, miRNA target enrichment, hierarchical clustering, host gene and *HOX* gene analyses, PCA analysis, and gene expression signature calculations.

## Supporting Information

Figure S1 **Schematic to illustrate the sPCC method.**
(PDF)

Figure S2 **For each of the** ***HOX*** **gene clusters the expression of the hosted miRNA best correlates with the expression of the** ***HOX*** **genes in that cluster.** The sPCC values for the genes in each *HOX* gene cluster were cumulated for the 136 miRNAs and plotted by ranking 136 cumulative sPCC values from highest to lowest. For each *HOX* cluster the top ten miRNAs are listed in a table.
(PDF)

Figure S3 **Cluster analysis of 136 miRNAs based on a pairwise comparison of negatively correlating genes using the sPCC method.** The miRNAs were divided into 17 functional clusters (I–XVII). Stippled red line: threshold of 12.5% of groups that defined the 17 clusters.
(PDF)

Figure S4 **miRNAs that are most epithelial and most mesenchymal in nature according to their positively correlated genes.** (*A*) Ranking of 136 miRNAs according to their correlation with the expression of either E or M genes calculated by taking sPCCs related to the average of EMT signatures 1–3 (see Figure 5). All 5 *miR-200* family members (shown in red) were found in the top 9 miRNAs most positively correlated with the expression of E genes (left stippled line). Additional E related miRNAs, *miR-203*, *miR-7*, *miR-375* and miR-425* are shown in orange. The miRNAs best correlating with the expression of M genes are labeled in blue (defined by stippled line on the right). (*B*) Overlap in positively correlated genes between the entire *miR-200* family and the miRNAs that are most epithelial in nature (red/orange) or most mesenchymal in nature (blue).
(PDF)

Figure S5 **Principal Component Analysis of 136 miRNAs according to positively correlating genes, with the two PCs (PC1 and PC2) with the highest score plotted.** The epithelial subcluster of miRNAs that regulated E-cadherin (see Figure 6) is labeled by a red circle.
(PDF)

Figure S6 **Correlation of miRNA expression with that of ribosomal protein genes.** The 136 miRNAs that correlated in their expression with those of ribosomal genes (RPs) were ranked according to the number of RPs that had positive or negative sPCC values with individual miRNAs. Rank order was determined by the factor: [number of positively correlating RPs] - [number of negatively correlating RPs].
(PDF)

Table S1 **Expression of miRNAs in 59 of NCI60 cell lines sorted according to numbers of cell lines with detectable expression.** miR-429 was found to be significantly expressed in more than 30 cell lines (data not shown).
(XLS)

Table S2 **miRNA seed families represented in the Q data set. miRNAs in red = family members for which no data sets were available.**
(XLS)

Table S3 **Analysis of the top 500 conserved genes predicted by TargetScan in the human genome to be targeted by one of the 136 miRNAs.**
(XLS)

Table S4 **Identification of miRNAs with tissue specific expression. FDR: False Discovery Rate.**
(XLS)

Table S5 **miRNAs that are part of either seed families, gene clusters or are tissue specifically expressed.**
(XLS)
