# Supplementary figures and images for: miRConnect: Identifying Effector Genes of miRNAs and miRNA Families in Cancer Cells
Source: PLoS One. 2011 Oct 26;6(10):e26521. doi: 10.1371/journal.pone.0026521 (PMC3202536; doi:10.1371/journal.pone.0026521)

Figure S1

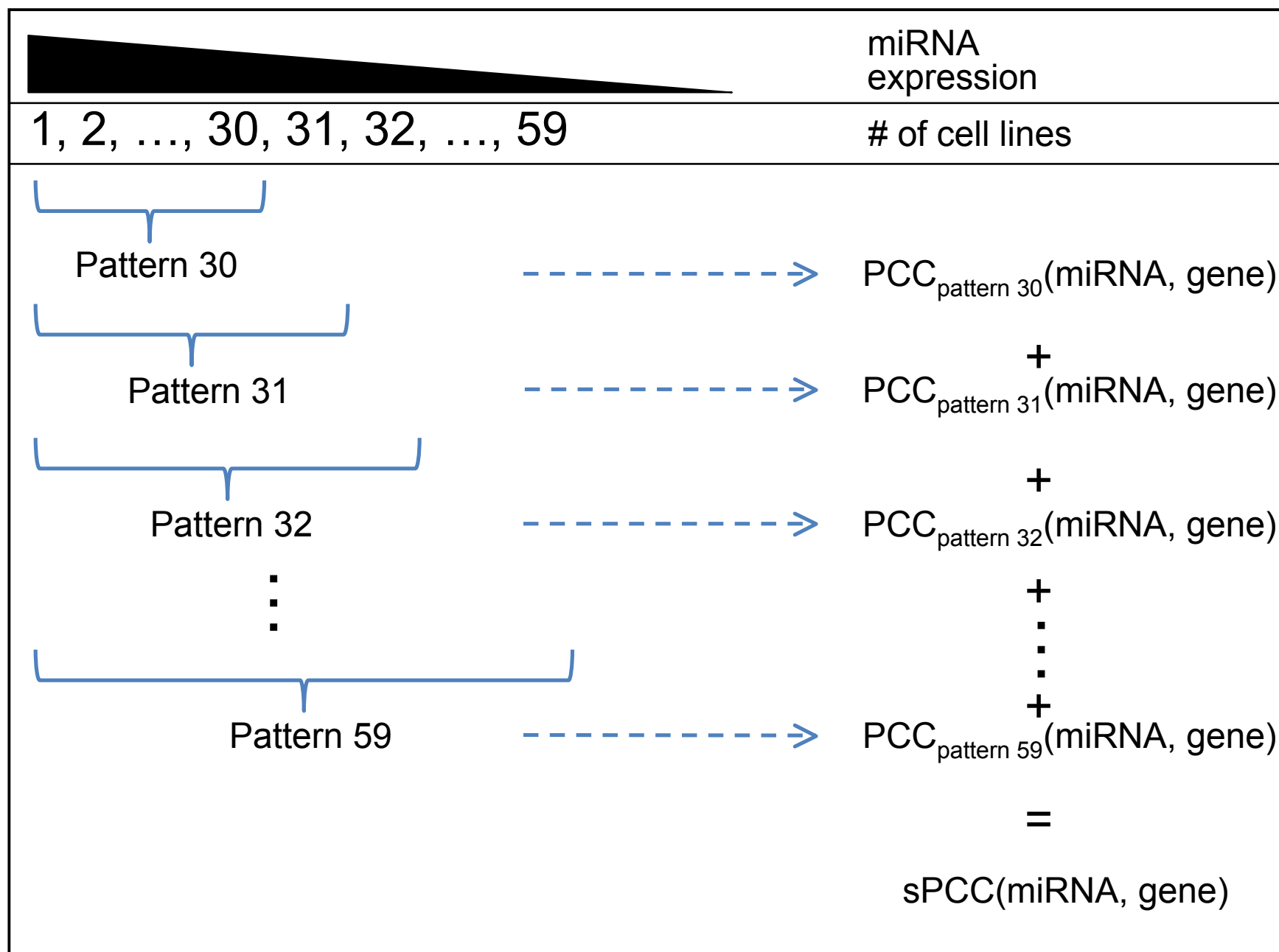

Supplement: Figure S1 — Schematic to illustrate the sPCC method. (PDF) [file pone.0026521.s001.pdf]

Figure S2

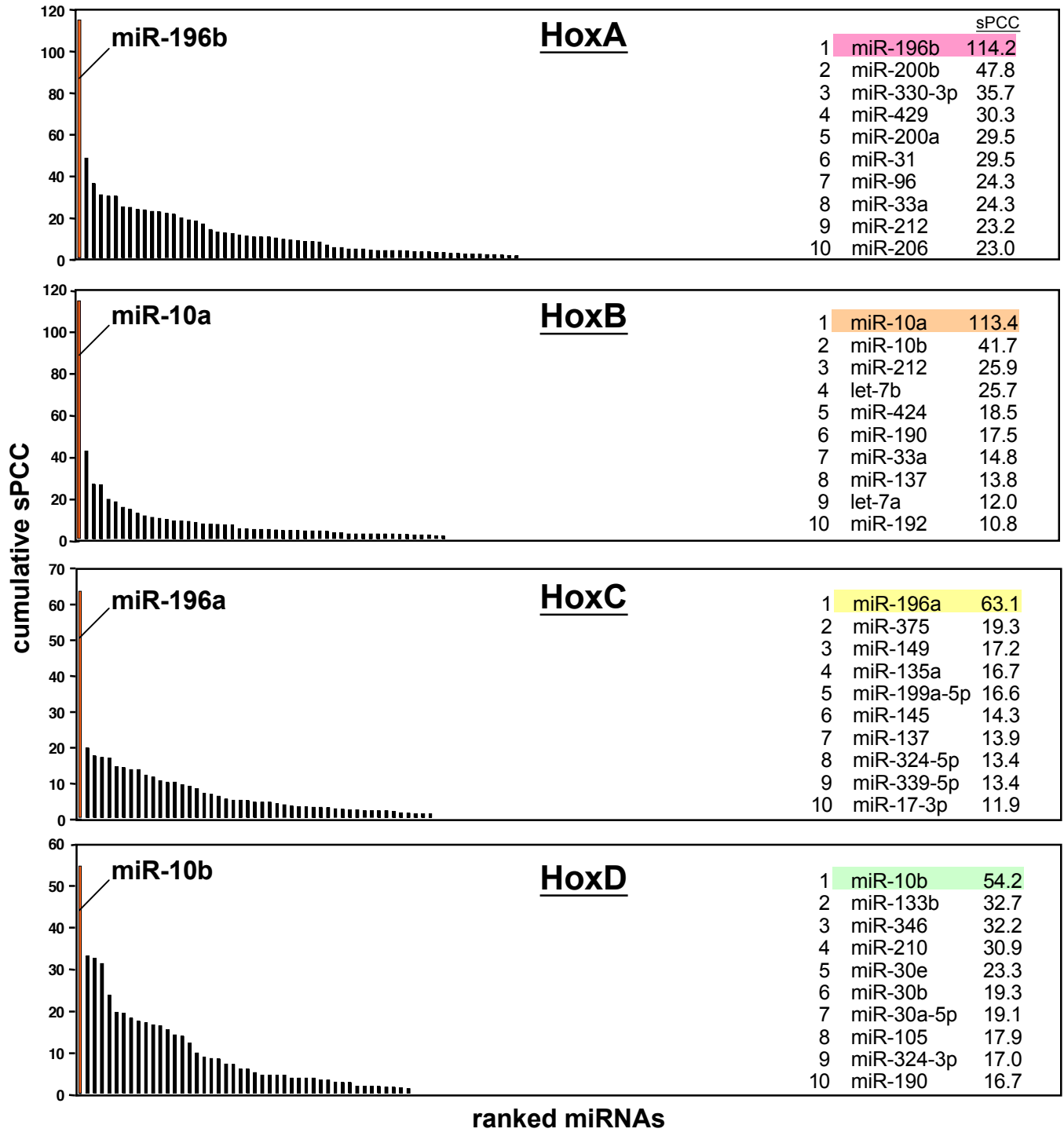

Supplement: Figure S2 — For each of the HOX gene clusters the expression of the hosted miRNA best correlates with the expression of the HOX genes in that cluster. The sPCC values for the genes in each HOX gene cluster were cumulated for the 136 miRNAs and plotted by ranking 136 cumulative sPCC values from highest to lowest. For each HOX cluster the top ten miRNAs are listed in a table. (PDF) [file pone.0026521.s002.pdf]

Figure S3

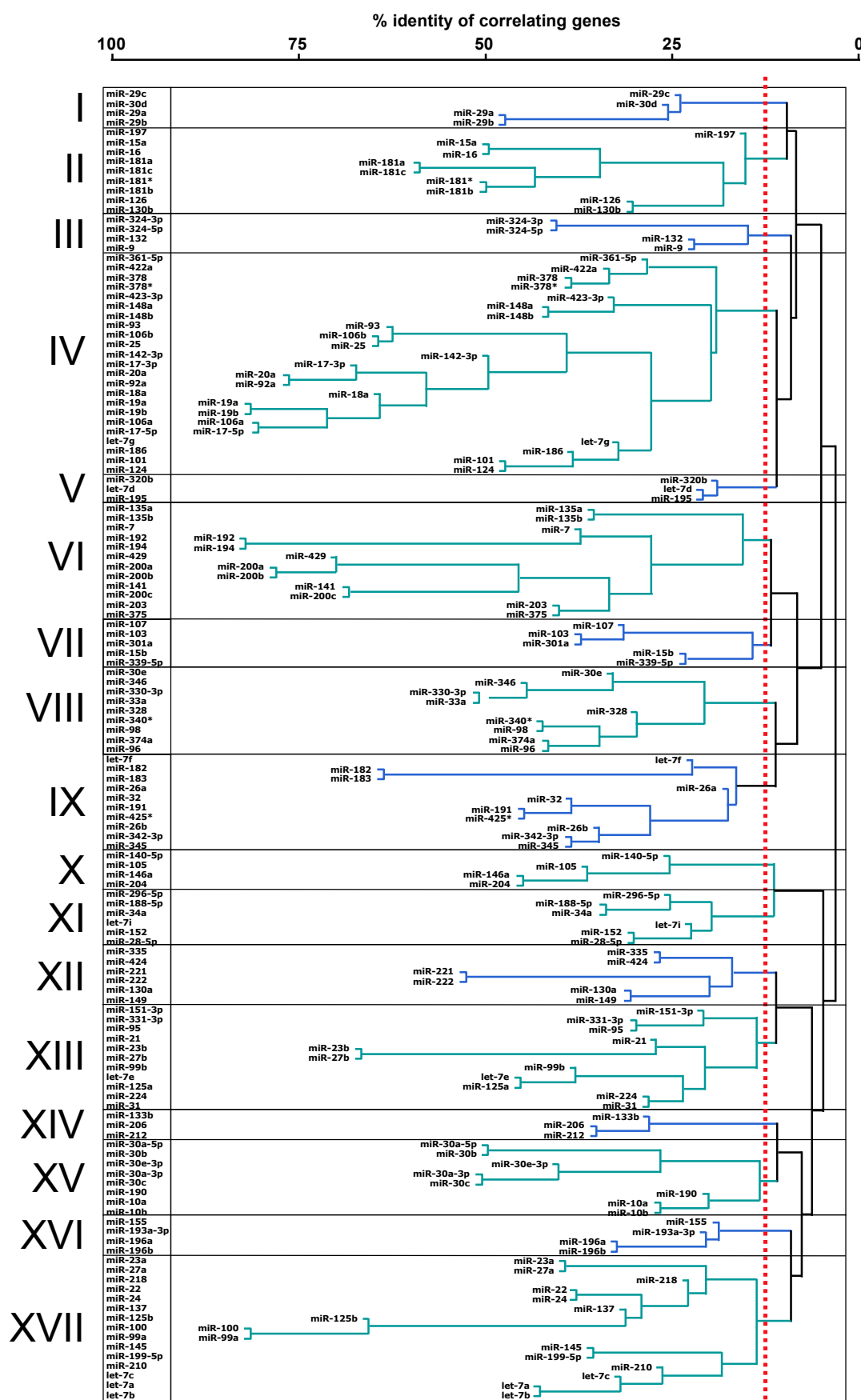

Supplement: Figure S3 — Cluster analysis of 136 miRNAs based on a pairwise comparison of negatively correlating genes using the sPCC method. The miRNAs were divided into 17 functional clusters (I–XVII). Stippled red line: threshold of 12.5% of groups that defined the 17 clusters. (PDF) [file pone.0026521.s003.pdf]

Figure S4

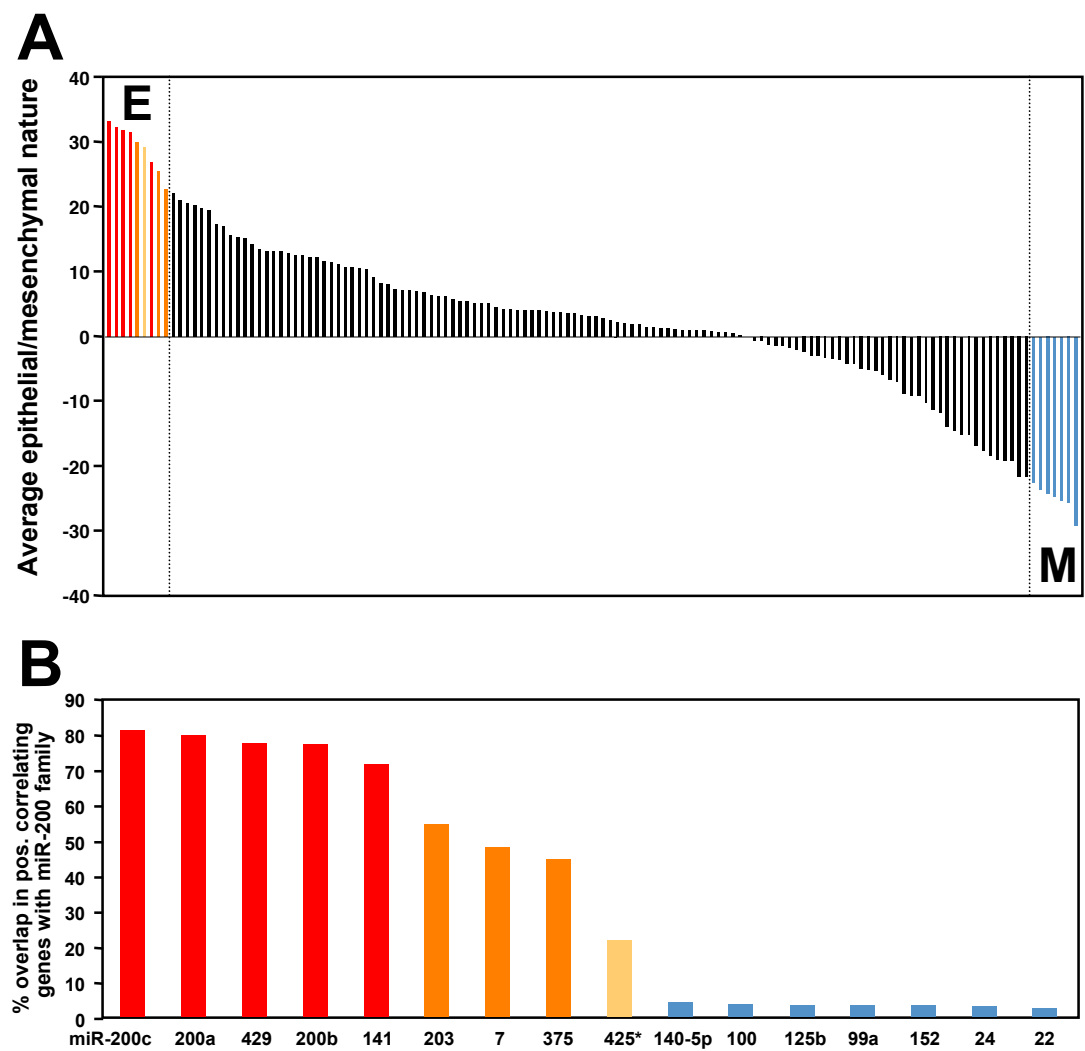

Supplement: Figure S4 — miRNAs that are most epithelial and most mesenchymal in nature according to their positively correlated genes. (A) Ranking of 136 miRNAs according to their correlation with the expression of either E or M genes calculated by taking sPCCs related to the average of EMT signatures 1–3 (see Figure 5). All 5 miR-200 family members (shown in red) were found in the top 9 miRNAs most positively correlated with the expression of E genes (left stippled line). Additional E related miRNAs, miR-203, miR-7, miR-375 and miR-425* are shown in orange. The miRNAs best correlating with the expression of M genes are labeled in blue (defined by stippled line on the right). (B) Overlap in positively correlated genes between the entire miR-200 family and the miRNAs that are most epithelial in nature (red/orange) or most mesenchymal in nature (blue). (PDF) [file pone.0026521.s004.pdf]

Figure S5

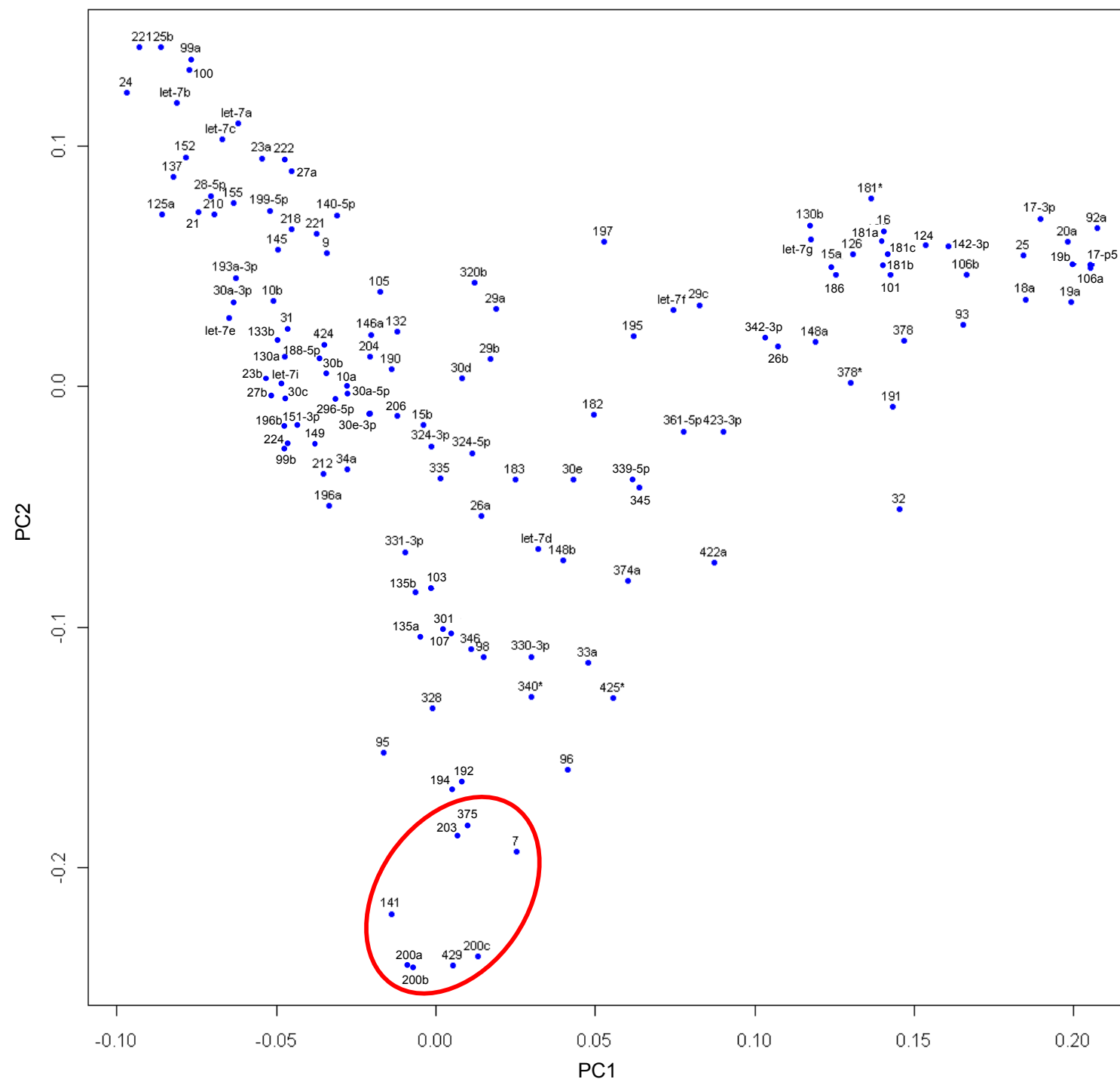

Supplement: Figure S5 — Principal Component Analysis of 136 miRNAs according to positively correlating genes, with the two PCs (PC1 and PC2) with the highest score plotted. The epithelial subcluster of miRNAs that regulated E-cadherin (see Figure 6) is labeled by a red circle. (PDF) [file pone.0026521.s005.pdf]

Figure S6

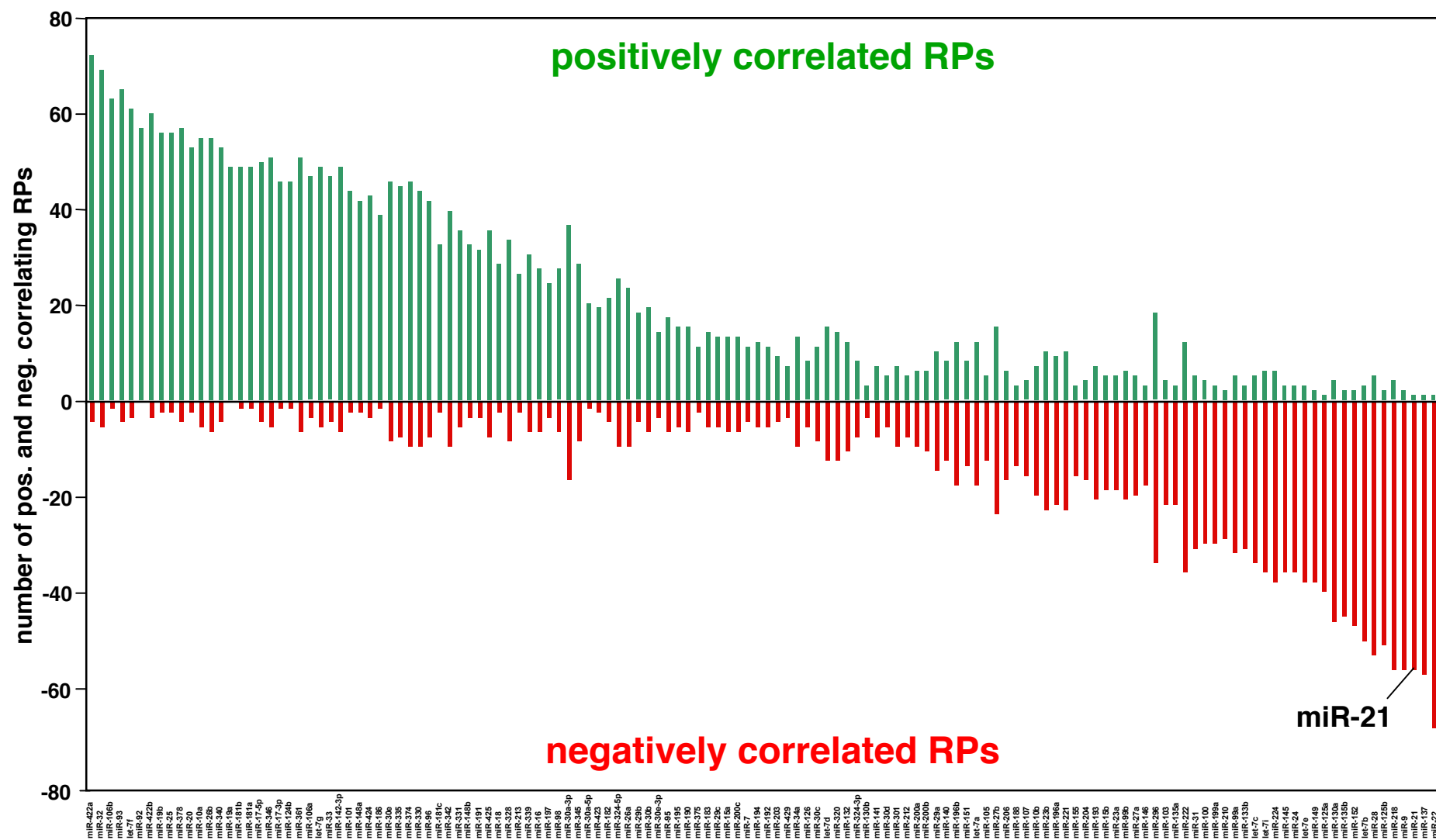

Supplement: Figure S6 — Correlation of miRNA expression with that of ribosomal protein genes. The 136 miRNAs that correlated in their expression with those of ribosomal genes (RPs) were ranked according to the number of RPs that had positive or negative sPCC values with individual miRNAs. Rank order was determined by the factor: [number of positively correlating RPs] - [number of negatively correlating RPs]. (PDF) [file pone.0026521.s006.pdf]
